# Copeptin is independently associated with vascular calcification in chronic kidney disease stage 5

**DOI:** 10.1186/s12882-020-1710-6

**Published:** 2020-02-07

**Authors:** Edyta Golembiewska, Abdul Rashid Qureshi, Lu Dai, Bengt Lindholm, Olof Heimbürger, Magnus Söderberg, Torkel B. Brismar, Jonaz Ripsweden, Peter Barany, Richard J. Johnson, Peter Stenvinkel

**Affiliations:** 1 Division of Renal Medicine and Baxter Novum, Department of Clinical Science, Intervention and Technology, Karolinska Institutet, Campus Flemingsberg, Stockholm, Sweden; 2 Department of Nephrology, Transplantology and Internal Medicine, Pomeranian Medical University, Al. Powstancow Wlkp. 72, 70-111 Szczecin, Poland; 3 Cardiovascular, Renal and Metabolism Safety, Clinical Pharmacology & Safety Sciences R&D, AstraZeneca, Gothenburg, Sweden; 4 Division of Medical Imaging and Technology, Department of Clinical Science, Intervention and Technology, Karolinska Institutet, Campus Flemingsberg, Stockholm, Sweden; 5 Division of Renal Diseases and Hypertension, School of Medicine, University of Colorado, Denver, CO USA

**Keywords:** Copeptin, Chronic kidney disease, End-stage renal disease, Vascular calcification

## Abstract

**Background:**

Vascular calcification (VC) is an independent predictor of cardiovascular disease (CVD) present in 30–70% of patients with chronic kidney disease (CKD). Copeptin is a sensitive surrogate marker of arginine vasopressin (AVP), which is involved in many pathophysiologic processes in CKD. The aim of the present study was to explore the association of copeptin with VC in CKD stage 5.

**Methods:**

Copeptin was investigated in conjunction with living donor kidney transplantation in 149 clinically stable CKD stage 5 patients (CKD5), including 53 non-dialyzed (CKD5-ND) and 96 dialysis patients treated by peritoneal dialysis (PD) (*n* = 43) or hemodialysis (HD) (*n* = 53). We analyzed the association of copeptin with presence and extent of VC ascertained both histologically in biopsies from the inferior epigastric artery (*n* = 137) and by coronary artery calcification (CAC) score measured by computed tomography.

**Results:**

Patients with higher copeptin were older, had higher systolic blood pressure, higher prevalence of CVD and their preceding time on chronic dialysis was longer. In Spearman’s rank correlations (Rho), copeptin concentrations were significantly associated with CAC score (Rho = 0.27; *p* = 0.003) and presence of medial VC (Rho = 0.21; *p* = 0.016). Multivariate logistic regression analysis showed that 1-SD higher age, male gender, diabetes and 1-SD higher copeptin were significantly associated with the presence of moderate-extensive VC.

**Conclusions:**

High circulating levels of copeptin in CKD5 patients are independently associated with the degree of medial calcification ascertained by histology of arterial biopsies. Thus, plasma copeptin may serve as a marker of the uremic calcification process.

## Background

Chronic kidney disease (CKD) is associated with markedly increased morbidity and mortality which is mainly a result of premature cardiovascular disease (CVD) [1–3]. The causes for the high prevalence of CVD are still not fully elucidated and cannot be explained only by traditional Framingham risk factors, such as age, hypertension, diabetes mellitus, dyslipidemia and smoking. In addition, CKD-specific risk factors, e.g. inflammation, prooxidant-antioxidant imbalance, protein-energy wasting, disorders in mineral metabolism and gut dysbiosis, also play a role [2, 4].

Medial vascular calcification (VC), i.e. calcific arteriosclerosis, an independent predictor of CVD, is reported in 30–70% of adult CKD patients, and ≈15% of pediatric CKD patients [5, 6]. VC develops in the intima together with atherosclerosis, which is defined as an increase in the deposition of plaques, and in the media together with arteriosclerosis, which is associated with vascular stiffening resulting from the loss of elasticity of muscle layer of the arterial wall. The presence of VC results in arterial dysfunction, such as NO-dependent endothelial dysfunction [7]. The extent of coronary artery calcification (CAC) examined by cardiac computed tomography (CT) is a useful tool in assessing extent of intima and medial VC [8, 9]. Medial calcification is predominantly related to abnormal metabolism in CKD and represents one of the uremic features entitled CKD – mineral and bone disorder (CKD-MBD) [10]. Although several biomarkers, such as calcium, phosphate, alkaline phosphatase, parathyroid hormone, fibroblast growth factor–23 (FGF-23), Klotho, vitamin D_3_, osteoprotegerin (OPG) and sclerostin, play important roles in pathological pathways of CKD-BMD [11–13], the exact pathogenic mechanisms remain unclear and additional factors are likely to be involved.

Recently, there is increased attention to the role of the arginine vasopressin (AVP) system, particularly AVP and copeptin, in the pathophysiological mechanisms of CKD. AVP, known also as antidiuretic hormone, is produced in the hypothalamus in the form of a precursor, pre-proAVP, and released from the posterior pituitary gland in response to different osmotic and non-osmotic forces. The principal role of AVP is the control of fluid balance by promoting water preservation by the kidney, but, in addition, AVP is involved in the mediation of arteriolar vasoconstriction and contributes to cardiovascular stability [14]. Copeptin, a 39-amino acid glycopeptide with a leucine-rich core segment and molecular mass of 5 kDa is derived from the C-terminal portion of the pre-provasopressin and is co-released with AVP in equimolar amounts. Copeptin is thought to be a reliable, sensitive and easy to measure surrogate marker of AVP [15]. This observation has prompted extensive research regarding copeptin – as a surrogate measure of AVP - in numerous clinical situations. High circulating copeptin is associated with decline in glomerular filtration rate and a greater risk of new-onset CKD [16, 17]. Moreover, high copeptin has been linked with increased risk of myocardial infarction, heart failure, hypertension, ischemic stroke, diabetes mellitus and metabolic syndrome [14, 18]. In patients with type 1 diabetes, elevated copeptin levels were strongly related to diabetic CKD and coronary atherosclerosis [19].

The aim of the present study was to examine the association of copeptin with the presence and extent of CAC (by CT) and medial VC ascertained in biopsies from the inferior epigastric artery in CKD5 patients at the time of living donor kidney transplantation (LD-Rtx).

## Methods

### Patients and study design

Circulating concentrations of copeptin were measured in clinically stable CKD 5 patients (*n* = 149) comprising non-dialyzed patients (CKD5-ND; *n* = 53), and dialysis patients (*n* = 96) treated by peritoneal dialysis (PD; *n* = 43) or hemodialysis (HD; *n* = 53) recruited from an ongoing study on vascular changes before undergoing LD-Rtx [13]. Their age ranged between 19 and 75 years, 101 (68%) were men, 16 (11%) had diabetes mellitus and 23 (15%) had previously been diagnosed with cerebrovascular, cardiovascular, and/or peripheral vascular disease (grouped as CVD).

The characteristics of patients, according to tertiles of copeptin, are shown in Table 1. Patients were recruited from March 2009 to February 2017. Exclusion criterion was unwillingness to participate. Informed consent to participate in the study – including consent to biopsy of inferior epigastric artery during transplantation procedure - was obtained from each patient. The Ethics Committee of the Karolinska Institutet (EPN) approved study protocols. The studies were conducted in adherence to the Declaration of Helsinki.

**Table 1:** Characteristics of CKD 5 patients according to tertiles of copeptin

	Low (*n* = 49)	Middle (*n* = 49)	High (*n* = 51)	*P* value
General characteristics				
Copeptin (pg/mL)	290 (216–328)	380 (344–438)	520 (455–677)	**0.001**
Age (years)	45 (24–62)	46 (24–65)	51 (29–67)	**0.030**
Males, n (%)	32 (65)	33 (67)	36 (71)	0.840
Diabetes mellitus, n (%)	4 (8)	5 (10)	7 (13)	0.663
Cardiovascular disease, n (%)	3 (6)	8 (16)	12 (23)	**0.041**
Previously on dialysis, n (%)	26 (53)	28 (57)	42 (82)	**0.015**
Dialysis vintage, years	0.2 (0–3.4)	0.3 (0–3.11)	1.0 (0–3.1)	**0.005**
Systolic BP, (mmHg)	133 (116–156)	146 (125–170)	144 (115–180)	**0.008**
Diastolic BP, (mmHg)	80 (67–96)	84 (72–95)	82 (67–100)	0.542
Nutritional status				
Body mass index, (kg/m^2^)	24.4 (21–29.7)	24.5 (20.5–29.9)	25.0 (20.9–29.8)	0.824
Lean body mass index, (kg/m^2^)^a^	18.3 (15.3–20.3)	18.6 (14.1–22.1)	18.2 (14.8–21)	0.888
Fat body mass index, (kg/m^2^)^a^	5.9 (3.4–10.1)	6.6 (3.6–10.7)	6.1 (3.1–10.1)	0.464
Markers of metabolism and nutrition				
Hemoglobin, (g/L)^b^	110 (97–129)	112 (97–130)	114 (96–131)	0.724
Albumin, (g/L)	35.0 (31.0–40.0)	35.0 (30.0–40.0)	34 (27.4–39.8)	0.388
Glucose, (mmol/L)^c^	5.4 (4.3–9.2)	5.4 (4.4–7)	5.8 (4.5–8.2)	0.286
HbA1c, (%)^d^	33.0 (19.0–40.0)	33.5 (25.5–40.5)	33.0 (23.9–40.4)	0.666
Triglyceride, (mmol/L)	1.2 (0.6–2.3)	1.3 (0.6–2.3)	1.4 (0.7–2.6)	0.072
Total cholesterol, (mmol/L)	4.5 (3.2–5.9)	4.4 (3.2–6.2)	4.2 (3.0–6.3)	0.661
HDL cholesterol, (mmol/L)	1.4 (1.0–2.0)	1.4 (0.8–2.2)	1.3 (0.8–2.0)	0.408
LDL cholesterol, (mmol/L)	2.7 (1.6–4.4)	2.6 (1.4–4.5)	2.6 (1.4–4.6)	0.916
Plasma osmolality (mmol/kg)	300 (290–315)	306 (288–319)	302 (290–314)	0.241
Creatinine (μmol/L)	661 (484–1086)	683 (451–1021)	829 (560–1136)	**0.006**
Biomarkers of inflammation				
hsCRP, (mg/L)	0.8 (0.2–9.0)	0.8 (0.2–3.9)	1.0 (0.2–7.0)	0.883
IL-6, (pg/mL)^e^	1.0 (0.1–6.0)	1.4 (0.1–5.7)	1.4 (0–6.5)	0.820
TNF (pg/mL)^f^	9.0 (7.2–15.2)	10.8 (8.3–15.2)	10.5 (7.6–21)	0.094
Medications				
β-blockers, n (%)	14 (27)	31 (63)	41 (80)	**< 0.0001**
Ca-blocker, n (%)	24 (45)	26 (53)	27 (53)	0.899
ACEi/ARB, n (%)	32 (65)	32 (65)	25 (49)	0.157
Statins, n (%)	15 (31)	16 (33)	21 (41)	0.499
Calcium-phosphate binders, n (%)	24 (49)	28 (57)	26 (51)	0.700
Biomarkers of mineral-bone disease and vascular calcification				
Calcium, (mmol/L)	2.3 (2.1–2.6)	2.3 (2.0–2.5)	2.3 (2.0–2.5)	0.814
Phosphate, (mmol/L)	1.7 (1.1–2.1)	1.7 (1.0–2.5)	1.6 (1.0–2.4)	0.964
ALP, (U/L)^f^	57 (33–109)	60 (38–155)	67 (37–118)	0.505
PTH, (pg/mL)	231 (94–623)	255 (75–594)	269 (54–520)	0.903
FGF-23, (pg/mL)^g^	3534 (223–27,943)	2820 (706–38,863)	7486 (1088–83,186)	0.257
Klotho (pg/mL)^h^	317 (178–603)	380 (197–970)	330 (119–513)	0.220
25 (OH) vitamin D	38 (16–79.8)	35 (20–73)	33.5 (12–65.7)	0.309
Sclerostin (pg/mL)^i^	369 (198–939)	409 (247–682)	515 (271–894)	0.067
Troponin T (μg/L)	17.0 (5.0–53.6)	19.5 (3.6–71.6)	32.0 (1.2–68.0)	**0.036**
Total BMD (g/cm^2^)^f^	1.2 (1.0–1.3)	1.1 (0.9–1.3)	1.1 (0.8–1.3)	0.100
CAC score (AU)^j^	0 (0–700)	2 (0–1546)	38 (0–1946)	0.055
Medial calcification, n (%)^k^				**0.030**
0–1	34 (43)	23 (29)	22 (28)	
2–3	13 (22)	20 (35)	25 (43)	

Continuous variables are presented as median (10–90 percentile). Categorical variables are presented as number (n)/percentage (%). Abbreviations: *Systolic BP* Systolic blood pressure, *Diastolic BP* Diastolic blood pressure, *HDL* High-density lipoprotein, *LDL* Low-density lipoprotein, *hsCRP* High-sensitivity C-reactive protein, *IL-6* Interleukin-6, *TNF* Tumor necrosis factor, *ACEi* Angiotensin-converting enzyme, *ARB* Angiotensin 2 receptor blocker, *ALP* Alkaline phosphatase, *PTH* Parathyroid hormone, *FGF-23* Fibroblast growth factor – 23, *Total BMD* Total bone mineral density, *CAC score (AU)* Calcification score (Agatston units)

Measurements were available in following numbers of patients:

^a^*n* = 123, ^b^*n* = 120, ^c^*n* = 111, ^d^*n* = 129, ^e^*n* = 90, ^f^*n* = 77, ^g^*n* = 65, ^h^*n* = 109, ^i^*n* = 82, ^j^*n* = 115, ^k^ n = 137

Every entry written in boldface in the aforementioned tables is of statistical significance (*p* < 0,05)

### CKD5-ND patients

The causes of renal disease in CKD5-ND patients (*n* = 53) were chronic glomerulonephritis (*n* = 27), renovascular disease or hypertension (*n* = 2), diabetic nephropathy (*n* = 1), other causes or unknown etiology (*n* = 23).

### PD patients

Prevalent PD patients (*n* = 43) had been treated with biocompatible fluids: glucose-based, amino acid-based, or, for the long dwell, icodextrin-based solutions. Their median dialysis vintage time was 11.3 months. The causes of CKD were as follows: chronic glomerulonephritis (*n* = 14), renovascular disease or hypertension (n = 1), diabetic nephropathy (*n* = 6), other causes or unknown etiology (*n* = 22).

### HD patients

Prevalent HD patients (*n* = 53) with median dialysis vintage time of 12.8 months were treated by conventional maintenance HD or other dialytic techniques. The causes of CKD were as follows: chronic glomerulonephritis (*n* = 15), renovascular disease or hypertension (*n* = 5), diabetic nephropathy (*n* = 3), and others or unknown etiology (*n* = 30).

### Biochemical measurements

Biochemical measurements of plasma high-sensitivity C-reactive protein (hsCRP), albumin, creatinine, troponin, cholesterol, triglycerides, HDL-cholesterol, calcium, phosphate, alkaline phosphatase (ALP), parathyroid hormone (PTH), fibroblast growth factor 23 (FGF-23), Klotho, 25 (OH) vitamin D, were performed at the Clinical Chemical Laboratory of Karolinska University Hospital, Stockholm, Sweden. Human sclerostin was analyzed with ELISA kit from R&D systems (Abingdon, UK). LDL was calculated using the Friedewald formula: [(total cholesterol) - (high-density lipoprotein cholesterol) – (triglycerides/5)]. Tumor necrosis factor (TNF), interleukin-6 (IL-6) were analyzed by immunometric assays on an Immulite 1000 Analyzer (Siemens Healthcare Diagnostics, Los Angeles, CA, USA) using commercial kits. Plasma osmolality was measured using VAPRO Vapor Pressure Osmometer 5520 (Wescor, USA).

Blood samples for copeptin measurement were taken in patients following overnight fasting, in hemodialyzed patients samples were taken the day after HD session. Copeptin concentration was measured with a commercial enzyme immunoassay (Cloud-Clone Corp., Houston, USA) according to the manufacturer’s protocol. Briefly, samples were incubated at 37 °C with respective reagents. Optical density was read at 450 nm immediately. The minimum detectable dose of copeptin is typically <6.1 pg/mL. The intra-assay coefficient variation (CV) was <10% and the inter-assay CV was <12%.

### Nutritional assessments

Body mass index (BMI) was calculated as weight in kilograms divided by the square of height in meters. Lean body mass (LBM) and fat body mass (FBM) were calculated by anthropometry based on measurements of the thickness of biceps, triceps, subscapular and supra-iliac skinfold as described previously [20], using equations by Siri [21]. LBM index and FBM index were calculated according to Kyle et al. [22]. Bone mineral density (BMD) was determined by dual-energy X-ray absorptiometry (DXA).

### CAC score

Cardiac CT scans were performed using a 64-channel detector scanner (Lightspeed VCT; General Electric (GE) Healthcare, Milwaukee, WI). CAC scores were expressed in Agatston units, the protocol and measurements as described previously in detail [13]. Total CAC score was calculated as the sum of CAC scores in the left main artery, the left anterior descending artery, the left circumflex artery, and the right coronary artery.

### Vascular scoring by histology

Inferior epigastric arteries were collected from patients within 20 min from the start of kidney transplantation procedure. After preparation, the sections were then stained with hematoxylin and eosin and von Kossa staining before evaluation by one experienced pathologist. The degree of medial calcification was quantified semiautomatically according to method described previously [13] and graded 0 to 3, where 0 indicated no calcification and 3 the highest degree of calcification. Patients graded as 0 and 1 represented no-minimal vascular calcification, and those graded 2 and 3 represented moderate-extensive vascular calcification.

### Statistical analyses

Continuous data are expressed as median (10th to 90th percentile) and nominal data as percentage. *P* value was set at *p* < 0.05. For comparisons between three groups non-parametric Kruskal-Wallis ANOVA-test was used for continuous variables and Chi-square test was used for nominal variables. Associations between variables were determined using non-parametric Spearman rank correlation analysis. Multivariate linear regression analyses of copeptin were used and results were shown as standardized β regression coefficients. We performed multinomial logistic regression analysis to examine factors associated for determinants of vascular calcification 0 and 1 score versus 2 and 3. Statistical analyses were performed using statistical software SAS version 9.4 (SAS Campus Drive, Cary, NC, USA) and Stata 16 (Stata Corporation, College Station, TX, USA).

## Results

Demographics and clinical characteristics of the examined patients according to copeptin tertiles are shown in Table 1. Patients with higher copeptin were older, had higher systolic blood pressure and were more frequently diagnosed with CVD. In addition, higher percentage of these patients had been undergoing dialysis with longer vintage. Characteristics of the studied CKD5 patients according to dialysis dependence are presented in Additional file 1: Table S1. There was no difference in copeptin concentration between PD and HD patients (427 vs. 412 pg/mL, *p* = 1.0), while plasma copeptin was significantly lower in CKD5-ND patients compared to PD and HD patients (351 vs. 427 pg/mL, *p* = 0.02; and 351 vs. 412 pg/mL, *p* = 0.001, respectively). Plasma osmolality and copeptin correlated in CKD5-ND patients (Rho = 0.28; *p* = 0.04), whereas in the two groups of dialyzed patients this association was not observed. In Spearman’s rank correlations (Rho), copeptin concentrations were significantly associated with age (Rho = 0.21; *p* = 0.011), presence of CVD (Rho = 0.20; *p* = 0.013), systolic blood pressure (Rho = 0.20; *p* = 0.01), triglycerides (Rho = 0.22; *p* = 0.007), serum creatinine (Rho = 0.26; *p* = 0.001), TNF (Rho = 0.27; *p* = 0.016), sclerostin (Rho = 0.22; *p* = 0.049), troponin T (Rho = 0.27; *p* = 0.001), CAC score (Rho = 0.27; *p* = 0.003) and histological scoring of medial calcification (Rho = 0.21; *p* = 0.016). Copeptin did not have significant association with gender and diabetes mellitus.

When CAC scores were categorized as CAC = 0, CAC ≥ 100 and CAC ≥ 1000, we found no statistically significant differences in copeptin concentration between the group of CAC = 0 and CAC ≥ 100 (372.9 [range 259.4–529.8] vs 426.8 [range 242.3–651.8], *p* = 0.76) but there was statistically significant difference between the group of CAC = 0 and CAC ≥ 1000 (372.9 [range 259.4–529.8] vs 453.9 [range 333.4–681.1], p = 0.001).

**Fig. 1:**
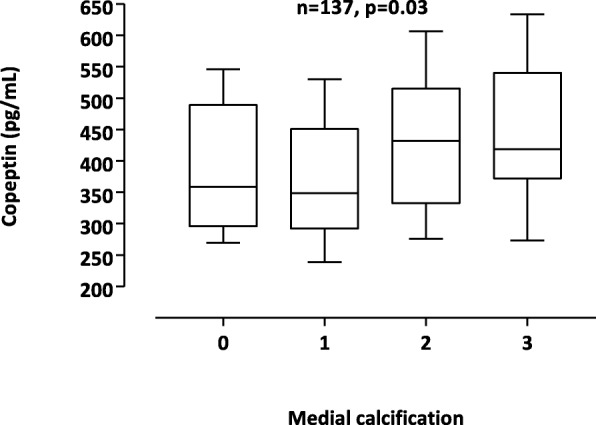
Plasma copeptin levels in relation to medial calcification

A multivariate linear regression model for determinants for plasma copeptin showed that 1-SD increase of age, 1-SD increase of serum creatinine and 1-SD increase of triglycerides were associated with higher plasma copeptin levels (Table 2). Patients with histological signs of moderate-extensive VC had significantly higher copeptin levels (Fig. 1).

**Table 2:** Multivariate linear regression for the prediction of copeptin, pg/ml (*n* = 149), expressed as standardized β-coefficients with other variables

	Copeptin (adj r^2^ = 0.14)
Parameters	standardized β (p value)
1-SD increase of age, years	**0.25 (0.002)**
Gender (male, female)	−0.08 (0.31)
1-SD increase of serum creatinine (μmol/l)	**0.26 (0.003)**
1-SD increase of triglyceride (mmol/L)	**0.16 (0.03)**
Systolic blood pressure (mmHg)	0.14 (0.07)

Every entry written in boldface in the aforementioned tables is of statistical significance (*p* < 0,05)

Univariate analysis showed no significant correlations between biomarkers of mineral-bone disease and VC; however, a positive correlation between hsCRP and VC was found (Rho = 0.18, *p* = 0.03). Receiver operating characteristic (ROC) curves for prediction of VC showed that the area under the curve (AUC) was for copeptin – AUC 0.64 (*p* = 0.02) and for hsCRP - AUC 0.54 (p = 0.02). Multivariate logistic regression analysis showed that 1-SD increase of age, male gender, presence of diabetes and 1-SD increase of copeptin were significantly associated with the presence of moderate-extensive VC (Table 3).

**Table 3:** Multivariate logistic regression model for determinants of VC 0 and 1 score versus 2 and 3 (*n* = 137)

	VC (pseudo r^2^ = 0.29)
Parameters	Odds Ratio 95%CI (p value)
1-SD increase of age, years	**2.5 (1.5–4.1) (0.001)**
Gender (male, female)	**4.4 (1.6–11.1) (0.003)**
Diabetes (No, Yes)	**23.2 (2.5–210.5) (0.005)**
CKD5-ND vs HD	0.7 (0.4–1.8) (0.42)
CKD5-ND vs PD	0.5 (0.4–1.8) (0.42)
1-SD increase of hsCRP (mg/L)	0.7 (0.4–1.3) (0.254)
1-SD increase of Copeptin, (pg/ml)	**1.6 (1.1–2.6) (0.043)**

Every entry written in boldface in the aforementioned tables is of statistical significance (*p* < 0,05)

## Discussion

We report that in CKD5 patients, high plasma copeptin levels are associated with age, CVD, dialysis vintage, systolic blood pressure and serum creatinine. The chief finding of the study was that elevated copeptin levels were associated with biopsy-verified media calcification. Multivariate analysis showed that copeptin was related to extent of VC independently of age, male gender and the presence of diabetes.

Vascular calcification, viewed in the past as a passive process, is now recognized as an active phenomenon [23]. The mechanisms underlying increased VC in CKD are not fully explained, but they involve disorders in calcium and phosphorus metabolism, and disturbed homeostasis in pro- and anti-calcification factors. Recently, novel tests, such as T50 functional test, have been developed to help in the determination of calcification propensity in CKD5 patients [24]. It has also been shown that uremia induces the differentiation of vascular smooth muscle cells (VSMCs) – the most important component of the media of arteries – into osteoblast-like cells due to up-regulation of transcription factors, such as Runt-related transcription factor 2 (Runx2) and Msh homebox 2 (Msx2) and increased alkaline phosphatase activity [25]. These changes contribute to the calcification of VSMC, in a way similar to bone formation (i.e. ossification).

**Fig. 2:**
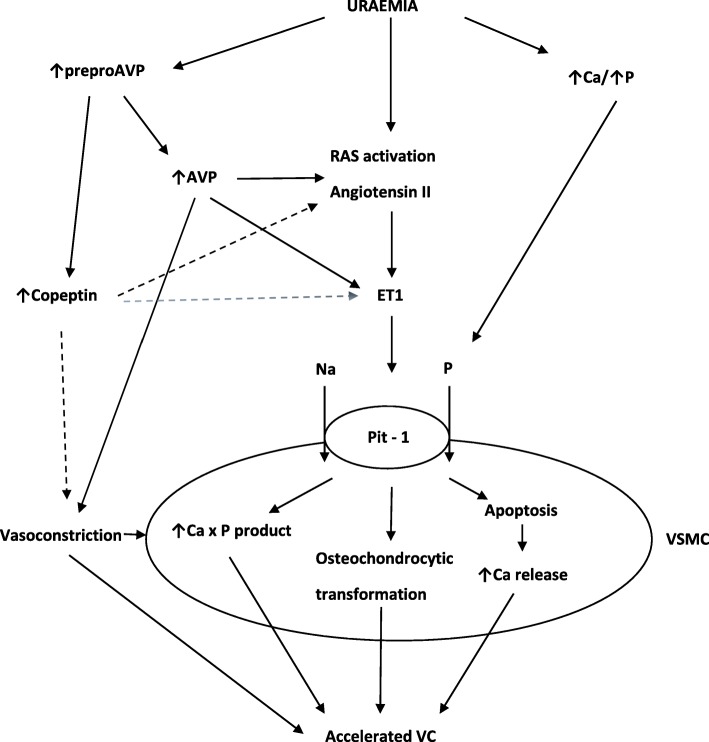
Mechanisms of vascular calcification in CKD. In the setting of uraemic milieu, activation of renin-angiotensin and vasopressin systems, upregulation of sodium-dependent phosphate transporter Pit-1 promotes osteochondrocytic transformation and apoptosis of vascular smooth muscle cell (VSMC) and, in consequence, accelerated vascular calcification. Dashed lines show possible copeptin links with the process

To the best of our knowledge, this is the first study to show a link between plasma copeptin level and the degree of VC in CKD. Emerging research suggest that vasopressin plays important role in the progression of CKD and hypertension via stimulation of the renin angiotensin aldosterone system (RAAS) leading to vasoconstriction and higher systemic and glomerular blood pressure [18]. Direct effects on VSMCs also result in vasoconstriction [26]. By modulating both the number and size of VSMC, AVP may play a direct role in the development of chronic vascular disease. It has been reported that AVP activates intracellular Ca^2+^ and protein kinase C (PC) via the activation of phospholipase (PL) C and D leading to augmentation of phosphate transport during proliferation of VSMC [27, 28]. Among other actions, AVP also stimulates secretion of endothelin-1 (ET-1) from endothelial cells which enhances endothelial dysfunction and coagulation disorders as well as increases cellular sodium-dependent phosphate transport via Pit1 and Pit2 [29, 30]. This suggests, that in the uremic hyperphosphatemic milieu, there is intensed entry of phosphate into VSMC. Furthermore, an increase in cellular phosphate can increase cellular calcification by different mechanisms: an increase in calcium x phosphate product, change of VSMC to a bone-producing cell phenotype and cell apoptosis that associates with increased secretion of pro-calcific factors [31]. A schematic presentation of mechanisms leading to VC including potential links with copeptin is shown in Fig. 2.

Our study showed positive correlation between plasma osmolality and copeptin in the group of non-dialyzed CKD5 patients, whereas such relationship was not observed in PD and HD groups, and non-dialyzed patients had also lower plasma copeptin levels than the other two groups. This accords with Ettema et al. [32] who examined copeptin levels in patients with different stages of CKD, including CKD5. They observed positive correlation between plasma osmolality and copeptin in patients with CKD and higher residual renal function (RRF) associated with lower plasma copeptin levels in HD patients which underscore the importance of RRF in the clearance of copeptin. Finally, they observed that both vasopressin and copeptin were removed during HD (with higher dialyzer clearance rate of vasopressin). We recently [33] reported that in patients undergoing PD, dialysate copeptin was positively correlated with plasma copeptin, indicating that also peritoneal clearance of copeptin may contribute to explain why there was no association between plasma copeptin and osmolality among the dialyzed patients in the current study.

A noteworthy finding of the study is the strong correlation of plasma copeptin with the use of β-blockers. To the best of our knowledge, such relationship has not been described before. Since the mechanism (s) of this correlation remain unclear, further studies are needed to clarify if this is merely a reflection of confounding by indication or if β-blockers do affect copeptin.peter.

The results of the present study should be considered given some important limitations. First, we examined CKD5 patients with different types of treatment; i.e. conservative, HD and PD. Moreover, since the group in which vascular biopsies were available was selected for LD-Rtx and thus represent a selected group of “healthier” CKD5 patients. Thus, since diabetic nephropathy was underrepresented compared to the typical CKD5 population the association between VC and copeptin may have been underestimated. Third, as in any observational study, cause-effect relations cannot be determined. Finally, although the circulating concentration of copeptin directly reflects that of AVP and copeptin shows the same response to osmotic changes as AVP in CKD, copeptin is reported to increase more than vasopressin suggesting that the clearance rate of copeptin is more markedly reduced than for vasopressin when GFR decreases. Thus, while copeptin reflects vasopressin levels, in CKD stages 4 and 5, a correction for renal function might be required [14, 32].

## Conclusions

High circulating levels of copeptin in CKD5 were found to associate with total CAC score (by cardiac CT), and with the degree of medial vascular calcification (by histology of arterial biopsies) even after adjusting for confounders. Further studies are needed to resolve if copeptin is simply a marker or a player in the complex uremic vascular calcification process.

## Supplementary information

**Additional file 1: Table S1**. Characteristics of CKD 5 patients stratified according to dialysis-dependence.

## Data Availability

The datasets used and and analysed during the current study are available from the corresponding author on reasonable request.

## Abbreviations

AVP: Arginine vasopressin
CAC: Coronary artery calcification
CKD: Chronic kidney disease
CVD: Cardiovascular disease
ET-1: Endothelin 1
HD: Hemodialysis
LD-Rtx: Living donor renal transplantation
PD: Peritoneal dialysis
VC: Vascular calcification
VSMC: Vascvular smooth muscle cell
