# Supplementary material for: Copeptin is independently associated with vascular calcification in chronic kidney disease stage 5
Source: BMC Nephrol. 2020 Feb 7;21:43. doi: 10.1186/s12882-020-1710-6 (PMC7006395; doi:10.1186/s12882-020-1710-6)
Supplement: Supplementary file 1 — Additional file 1: Table S1. Characteristics of CKD 5 patients stratified according to dialysis-dependence. [file 12882_2020_1710_MOESM1_ESM.docx]

**Table S1.** Characteristics of CKD 5 patients stratified according to dialysis-dependence.

|  | **Non-dialysis patients** | **Dialysis patients** | |  |
| --- | --- | --- | --- | --- |
|  | **CKD5-ND**  (n=53) | **HD**  (n=53) | **PD**  (n=43) | **P value** |
| ***General characteristics*** | | | | |
| Age (years) | 47 (24-64) | 48 (26-63) | 48 (23-69) | 0.857 |
| Males, n (%) | 36 (68) | 36 (68) | 36 (68) | 0.998 |
| Diabetes mellitus, n (%) | 3 (5) | 6 (11) | 7 (16) | 0.232 |
| Cardiovascular disease, n (%) | 5 (9) | 10 (19) | 8 (19) | 0.297 |
| Dialysis vintage, years | 0 | 1.1 (0.2-4.2) | 0.9 (0.2-3.4) | **<0.0001** |
| Systolic BP, (mmHg) | 145 (127-168) | 140 (110-179) | 135 (115-167) | 0.306 |
| Diastolic BP, (mmHg) | 85 (73-95) | 82 (65-96) | 82 (69-103) | 0.846 |
| ***Nutritional status*** | | | | |
| Body mass index, (kg/m^2^) | 24.6 (20.4-29.6) | 24.7 (20.8-31.9) | 24.5 (20.6-28.7) | 0.636 |
| Lean body mass index, (kg/m^2^)^a^ | 18.5 (15.0-20.8) | 18.0 (15.8-22.5) | 18.4 (14.3-21) | 0.987 |
| Fat body mass index, (kg/m^2^)^a^ | 6.3 (3.3-9.8) | 6.6 (2.9-11.7) | 6.0 (3.7-9.5) | 0.350 |
| ***Markers of metabolism and nutrition*** | | | | |
| Hemoglobin, (g/L)^b^ | 114 (100-129) | 114 (98-135) | 112 (94-136) | 0.619 |
| Albumin, (g/L) | 35.0 (28.3-42.0) | 36.0 (30.0-40.0) | 34 (29.0-38.0) | 0.084 |
| Glucose, (mmol/L)^c^ | 5.5 (4.2-8.3) | 6.1 (4.7-9.5) | 5.6 (4.7-9.6) | 0.210 |
| HbA1c, (%)^d^ | 34.0 (22.5-43.7) | 32.0 (20.0-40.7) | 37.0 (26.4-50.0) | **0.006** |
| Triglyceride, (mmol/L) | 1.3 (0.7-2.2) | 1.3 (0.7-2.7) | 1.2 (0.6-2.4) | 0.828 |
| Total cholesterol, (mmol/L) | 4.3 (3.0-5.5) | 4.1 (3.0-6.4) | 4.5 (3.2-6.0) | 0.282 |
| HDL cholesterol, (mmol/L) | 1.3 (0.9-1.8) | 1.4 (0.9-2.4) | 1.3 (0.8-2.1) | 0.406 |
| LDL cholesterol, (mmol/L) | 2.5 (1.6-3.9) | 2.4 (1.4-4.7) | 2.8 (1.4-4.4) | 0.287 |
| Plasma osmolality (mmol/kg) | 309 (298-320) | 299 (286-310) | 301 (288-313) | **<0.0001** |
| Creatinine (μmol/L) | 723 (440-1035) | 725 (519-1020) | 743 (523-1306) | **0.023** |
| ***Biomarkers of inflammation*** | | | | |
| hsCRP, (mg/L) | 0.9 (0.2-3.9) | 1.1 (0.2-8.6) | 0.7 (0.2-6.0) | 0.255 |
| IL-6, (pg/mL)^e^ | 0.8 (0-1.7) | 1.5 (0-11.9) | 1.7 (0.4-4.8) | **0.009** |
| TNF (pg/mL)^f^ | 9.5 (7.1-12.4) | 14.3 (7.6-26.6) | 10.1 (8.5-16.1) | **0.002** |
| ***Medications*** | | | | |
| β-blockers, n (%) | 26 (49) | 32 (60) | 28 (65) | 0.253 |
| Ca-blocker, n (%) | 33 (62) | 19 (36) | 25 (58) | **0.014** |
| ACEi/ARB, n (%) | 40 (75) | 26 (49) | 23 (53) | **0.011** |
| Statins, n (%) | 22 (41) | 15 (28) | 15 (35) | 0.359 |
| Calcium-phosphate binders, n (%) | 29 (55) | 26 (49) | 23 (54) | 0.830 |
| ***Biomarkers of mineral-bone disease and vascular calcification*** | | | | |
| Calcium, (mmol/L) | 2.3 (2.0-2.5) | 2.3 (2.0-2.5) | 2.3 (2.0-2.6) | 0.698 |
| Phosphate, (mmol/L) | 1.7 (1.2-2.4) | 1.7 (1.0-2.3) | 1.5 (1.0-2.4) | 0.430 |
| ALP, (U/L)^f^ | 57 (35-129) | 71 (40-114) | 59 (32-131) | 0.430 |
| PTH, (pg/mL) | 264 (84-595) | 264 (50-586) | 222 (85-525) | 0.551 |
| FGF-23, (pg/mL)^g^ | 2974 (590-31243) | 6331 (928-83853) | 3351 (804-72560) | 0.395 |
| Klotho (pg/mL)^h^ | 347 (161-668) | 338 (197-629) | 347 (194-544) | 0.765 |
| 25 (OH) vitamin D | 40 (25-72) | 38 (21-76) | 30 (9-60) | **0.001** |
| Sclerostin (pg/mL)^i^ | 399 (207-685) | 428 (238-930) | 442 (228-905) | 0.265 |
| Troponin T (μg/L) | 19.0 (6.0-55.0) | 24.5 (0.1-62.2) | 28.5 (5.0-88.8) | 0.322 |
| Total BMD (g/cm^2^)^f^ | 1.2 (1.0-1.3) | 1.1 (0.8-1.3) | 1.2 (1.0-1.3) | **0.005** |
| CAC score (AU)^j^  Medial calcification, n (%)^k^  0-1  2-3 | 2 (0-1124)  27 (34)  21 (36) | 11 (0-1041)  27 (34)  20 (34) | 12 (0-1932)  25 (32)  17 (30) | 0.639  0.492 |
| Copeptin (pg/mL) | 351 (238-508) | 412 (275-623) | 427 (266-585) | **0.005** |

Continuous variables are presented as median (10 – 90 percentile). Categorical variables are presented as number (n)/percentage (%). Abbreviations: Systolic BP, systolic blood pressure; Diastolic BP, diastolic blood pressure; HDL, high-density lipoprotein; LDL, low-density lipoprotein; hsCRP, high-sensitivity C-reactive protein; IL-6, interleukin-6; TNF, tumor necrosis factor; ACEi, angiotensin-converting enzyme; ARB, angiotensin 2 receptor blocker; ALP, alkaline phosphatase; PTH, parathyroid hormone; FGF-23, fibroblast growth factor – 23; Total BMD, total bone mineral density; CAC score (AU), calcification score (Agatston units).

Measurements were available in following numbers of patients:

**^a^** n=123, ^b^ n=120, ^c^ n=111, ^d^ n=129, ^e^ n=90, ^f^ n=77, ^g^ n=65, ^h^ n=109, ^i^ n=82, ^j^ n=115, ^k^ n=137.
